# Supplementary material for: FoxM1 repression during human aging leads to mitotic decline and aneuploidy-driven full senescence
Source: Nat Commun. 2018 Jul 19;9:2834. doi: 10.1038/s41467-018-05258-6 (PMC6053425; doi:10.1038/s41467-018-05258-6)
Supplement: Supplementary file 1 — Supplementary Information [file 41467_2018_5258_MOESM1_ESM.pdf]

Supplementary Information

Macedo et al., *FoxM1 repression during human aging leads to mitotic decline and aneuploidy-driven full senescence*

Supplementary Table 1. Fibroblasts from skin biopsies of Caucasian males with different ages used in this study (HDFs).

HDFs (age)	Reference, Repository	PDL at freeze	Cumulative PDL	PD (hrs)
N	C-0004-5C lot 74812, Invitrogen	n/a	X+1.7 / +3.2 / +8.2	40.3
N	DFM021711A, Zen Bio	n/a	X+2.2 / +4.6 / +7.2	41.2
8y	GM08398, Coriell Cell Repository	5.73	7.4 / 11.5 / 14.3	72.4
10y	GM03348, Coriell Cell Repository	4.2	5.9 / 7.8 / 9.9	49.1
22y	DFME102710, Zen Bio	n/a	X+1.8 / +2.2 / +3.0	49.5
22y	GM23976, Coriell Cell Repository	7.6	9.6 / 11.4 / 13.9 / 15.4	41.3
52y	GM28967, Coriell Cell Repository	4.17	5.9 / 7.4 / 8.9 / 10.4	55.0
54y	DFM062509, Zen Bio	n/a	X+1.73 / +3.1 / +4.4	114.8
84y	AG11488, Coriell Cell Repository	10	11.8 / 13.4 / 15.3 / 17	85.8
87y	AG10884, Coriell Cell Repository	7	8.8 / 10.9 / 12.1 / 13.3	119.1
8y, HGPS	AG03513, Coriell Cell Repository	15	16.9 / 18.4 / 19.9 / 21.7 / 23.3	111.1

All donors were reported as ‘healthy’, except the 8-year-old donor with the Hutchison-Gilford Progeria Syndrome (HGPS). Population doubling levels (PDL) at freeze and during experimental passages (≤5) are indicated. Average population doubling time (PD) during experimental passages is also indicated. PDL and PD were calculated as described in Materials and Methods. N, neonatal. n/a, not available.

Supplementary Table 2. Percentage of cells that deviate from modal chromosome number in fibroblast cell populations stained for FISH.

**HDF**	**Mode (% deviation) (Fig. 1a)**			
	**Chr 7**	**Chr 12**	**Chr 18**	**Chrs 7+12+18**
N/N	2 (0.344)$^{\#}$	2 (0.319)$^{\#}$	2 (0.270)$^{\#}$	6 (0.934)$^{\#}$
8/10y	2 (0.352)	2 (0.254)	2 (0.332)	6 (0.938)
22/22y	2 (0.481)	2 (0.449)	2 (0.224)	6 (1.154)
52/54y	2 (0.582)	2 (0.272)	2 (0.737)**	6 (1.590)*
84/87y	2 (0.922)***	2 (0.453)	2 (0.388)	6 (1.762)***
Prog	2 (0.646)	2 (0.109)	2 (0.437)*	6 (1.966)*

**HDF**	**Mode (% deviation) (Fig. 5d)**			
	**Chr 7**	**Chr 12**	**Chr 18**	**Chrs 7+12+18**
8/10y	2 (0.352)$^{\#}$	2 (0.254)$^{\#}$	2 (0.332)$^{\#}$	6 (0.938)$^{\#}$
10y siFoxM1	2 (0.863)**	2 (0.501)	2 (0.390)	6 (1.829)**

**HDF**	**Mode (% deviation) (Fig. 6e)**			
	**Chr 7**	**Chr 12**	**Chr 18**	**Chrs 7+12+18**
84/87y	2 (0.922)$^{\#}$	2 (0.453)$^{\#}$	2 (0.388)$^{\#}$	6 (1.762)$^{\#}$
84/87y FoxM1dNdK	2 (0.472)**	2 (0.236)$^{p=0.055}$	2 (0.177)*	6 (0.886)****

**HDF**	**Mode (% deviation) (Sup Fig.9e)**			
	**Chr 7**	**Chr 12**	**Chr 18**	**Chrs 7+12+18**
Prog	2 (0.668)$^{\#}$	2 (0.612)$^{\#}$	2 (0.550)$^{\#}$	6 (1.966)$^{\#}$
Prog FoxM1dNdK	2 (0.646)	2 (0.109)**	2 (0.437)	6 (1.192)$^{p=0.065}$

Chr, chromosome. >2500 nuclei were counted per chromosome per sample. # Cell population used as reference for statistical analysis (χ^2 test). * $p<0.05$; ** $p<0.005$; *** $p<0.001$; **** $p<0.0001$.

Supplementary Table 3. Primers used for qPCR.

Gene Name	Gene Symbol	Forward primer	Reverse primer	Length (bp)	Assay Design	Specie
Forkhead box M1	FOXM1	5'-ATACGTGGATTGAGGACCACT-3´	5'-TCCAATGTCAAGTAGCGGTTG-3´	175	Intron-spanning	Human
TATA box binding protein	TBP	5'-GAGCCAAGAGTGAAGAACAGTC-3´	5'GCTCCCCACCATATTCTGAATCT-3´	116	Intron-spanning	Human
Cyclin dependent kinase inhibitor 1A	CDKN1A/ p21	5'-TGGACCTGGAGACTCTCAGG-3´	5'-CGGATTAGGGCTTCCTCTTGG-3´	104	Intron-spanning	Human
C-X-C motif chemokine ligand 8	CXCL8/IL8	5'-GCCTTCCTGATTTCTGCAGCT-3´	5'-GCACTGACATCTAAGTTCTTTAGCA-3´	75	Intron-spanning	Human
Family with sequence similarity 214 member B	Fam214b	5'-CACCATCCAAGTGACCTTATTTAACC-3'	5'-AGAAGTCAAAGGTCACAAGGAACAT-3'	71	Intron-spanning	Human
Tetraspanin 13	TSPAN13	5'-CGCCATGTGCTCCAATCATAG-3´	5'-GTAGGTCAGCCAAACACCCA-3´	110	Intron-spanning	Human
Matrix metallopeptidase 1	MMP1	5'-AGCGTGTGACAGTAAGCTAACC-3´	5'-AACTTCCGGGTAGAAGGGATTTG-3´	109	Intron-spanning	Human
Forkhead box M1	FOXM1	5'-ATCGCTACTTGACATTGGACCA-3'	5'-GATTGGGTCGTTTCTGCTGTG-3´	96	Intron-spanning	Mouse
TATA box binding protein (Tbp)-associated factor, RNA polymerase I	TBP	5'-CTTCCTGCCACAATGTCACAG-3´	5'-CCTTTCTCATGCTTGCTTCTCTG-3´	118	Intron-spanning	Mouse

Supplementary Table 4. Primers used for cloning in pRetrox or pLVX vectors.

PCR fragment	PCR fragment name	Forward primer	Reverse primer
H2B-GFP-α-tubulin-mCherry	BglII-H2B-GFP-α-tubulin-mCherry-BamHI-NotI	5'-TATATAAGATCTATGCCAGAGCCAGCGAAGTCT-3´	5'-TATATAGCGGCCGCGGATCCCTACTGTAGCTCAGGAATAAAC-3´
FoxM1⁻ ΔNΔKEN	BglII-FoxM1⁻ ΔNΔKEN-BamHI-NotI	5'-TATATAAGATCTATGTGTCACCTGGAGCAGCGACAGG-3´	5'-TATATAGCGGCCGCGGATCCTTAGTATTCCTCTCCTTCTTCC-3´
H2B-GFP-T2A	BglII-H2B-GFP-T2A-BamHI-NotI	5'-TATATAAGATCTATGCCAGAGCCAGCGAAGTCT-3'	5'-ATAGCGGCCGCGGATCCGGGCCGGGGTTCTCCTCCACGTCGCCGCAGGTCAGCAGGGAGCCGCCCTCGCCGCCGGAGCCGCCGCCCTTGTACAGCTCGTCCATGCC-3'
mCherry	BglII-mCherry-BamHI-NotI	5'-TATATAAGATCTATGGTGAGCAAGGGCGAGGAG-3'	5'-TATATAGCGGCCGCGGATCCTCCTCCGGGTCCTCCCTTGTACAGCTCGTCCATGCC-3'
α-tubulin	BglII-α-tubulin-BamHI-NotI	5'-TATATAAGATCTATGCGTGAGTGCATCTCCATC-3'	5'-TATATAGCGGCCGCGGATCCTTAGTATTCCTCTCCTTCTTCC-3'
α-tubulin-mCherry	BglII-α-tubulin-mCherry-BamHI-NotI	5'-TATATAAGATCTATGGTGAGCAAGGGCGAGGAG-3'	5'-TATATAGCGGCCGCGGATCCTTAGTATTCCTCTCCTTCTTCC-3'

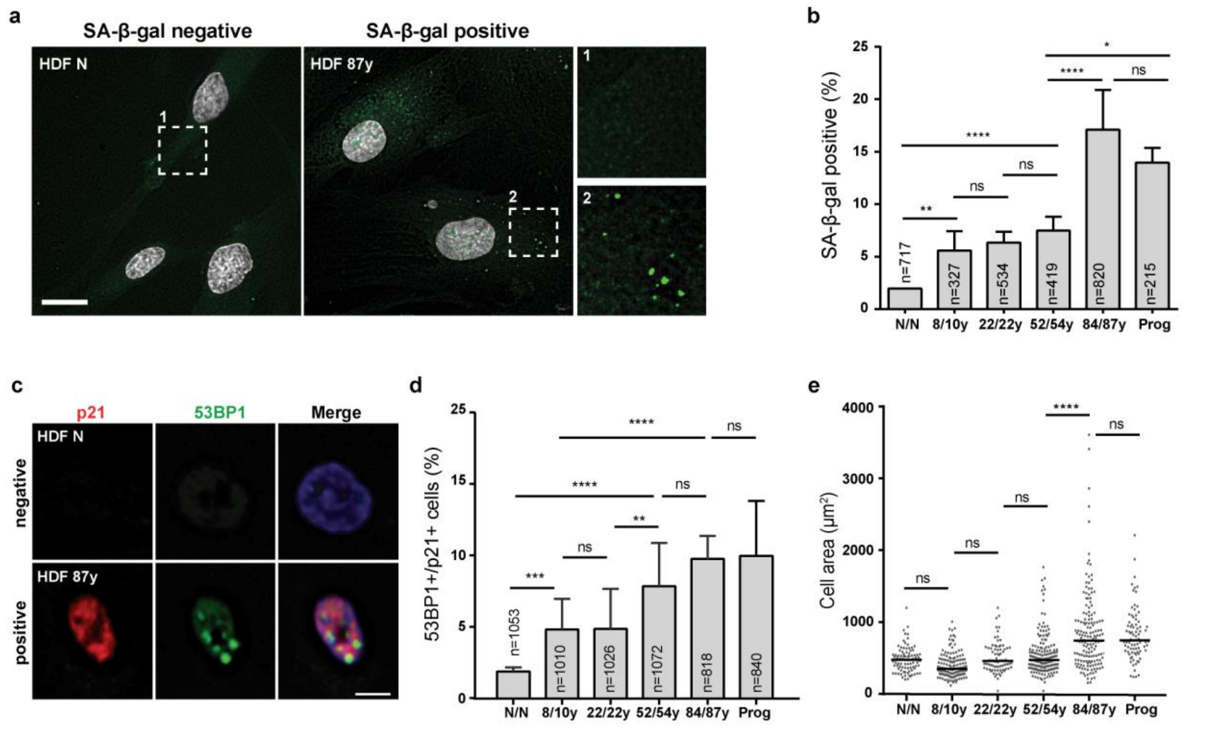

Supplementary Figure 1. Senescence markers in human dermal fibroblasts from different age donors. **a** Representative images of senescence-associated β-galactosidase (SA-β-gal) negative (HDF N) and positive (HDF 87y) cells. Insets are 2x magnifications. Scale bar, 20 µm. **b** Percentage of cells staining positive for SA-β-gal assay. **c** Representative images of cells negative (HDF N) and positive (HDF 87y) for double immunostaining of Cdkn1a/p21 (cell cycle inhibition) and 53BP1 (≥1 foci; DNA damage) senescence markers. Scale bar, 10 µm. **d** Percentage of Cdkn1a/p21 positive cells with 53BP1foci. **e** Cellular area as readout for senescence-associated enlarged cell morphology. Scatter plots show mean of n>50 cells. In all graphs, bars represent mean ± s.d. values from three independent experiments using two biological samples of similar age (except for progeria). Sample size (n) is indicated in each graph. ns: $p>0.05$, $*p\leq0.05$, $**p\leq0.01$, $***p\leq0.001$ and $****p\leq0.0001$ by two-tailed χ^2 (b, d) and Mann-Whitney (e) statistical tests.

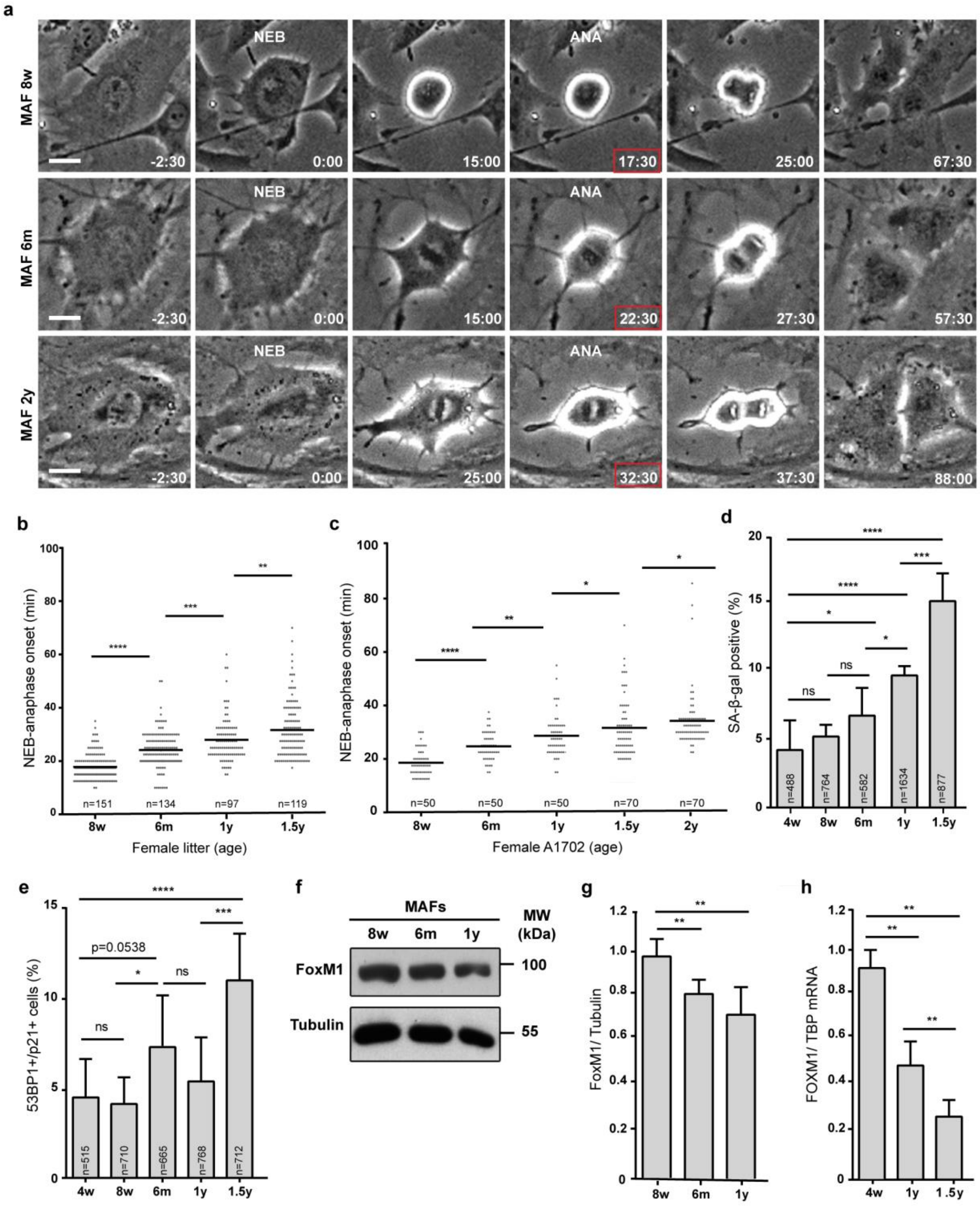

Supplementary Figure 2. Mitotic delay, cellular senescence and FoxM1 repression during mouse normative aging. **a** Movie frames of mitosis in MAFs collected at different age time-points as indicated. NEB, nuclear envelope breakdown. ANA, anaphase onset. Time min:sec. Scale bar, 20 µm. **b** Mitotic duration (NEB to anaphase onset) of individual MAFs collected

at different age time-points from ≥3 sv/129 sister females. **c** Mitotic duration of individual MAFs collected at different age time-points from one randomly chosen single female (Al702). **d** Percentage of cells staining positive for senescence-associated β-galactosidase assay. **e** Percentage of Cdkn1a/p21 positive cells with 53BP1 foci. **f** Western blot analysis of FoxM1 protein levels in mitotic extracts of different age MAF samples. **g** FoxM1 protein levels normalized to α-tubulin levels and 8w sample levels. **h** *FOXM1* transcript levels in total RNA from different age mitotic cell samples normalized to *TBP* transcript levels and compared to 4w sample. In all graphs, bars represent mean ± s.d. values from three independent experiments. Sample size (n) is indicated in each graph. ns: $p>0.05$, *$p\leq0.05$, **$p\leq0.01$, ***$p\leq0.001$ and ****$p\leq0.0001$ by two-tailed χ^2 (d, e) and Mann-Whitney (b, c, g and h) statistical tests.

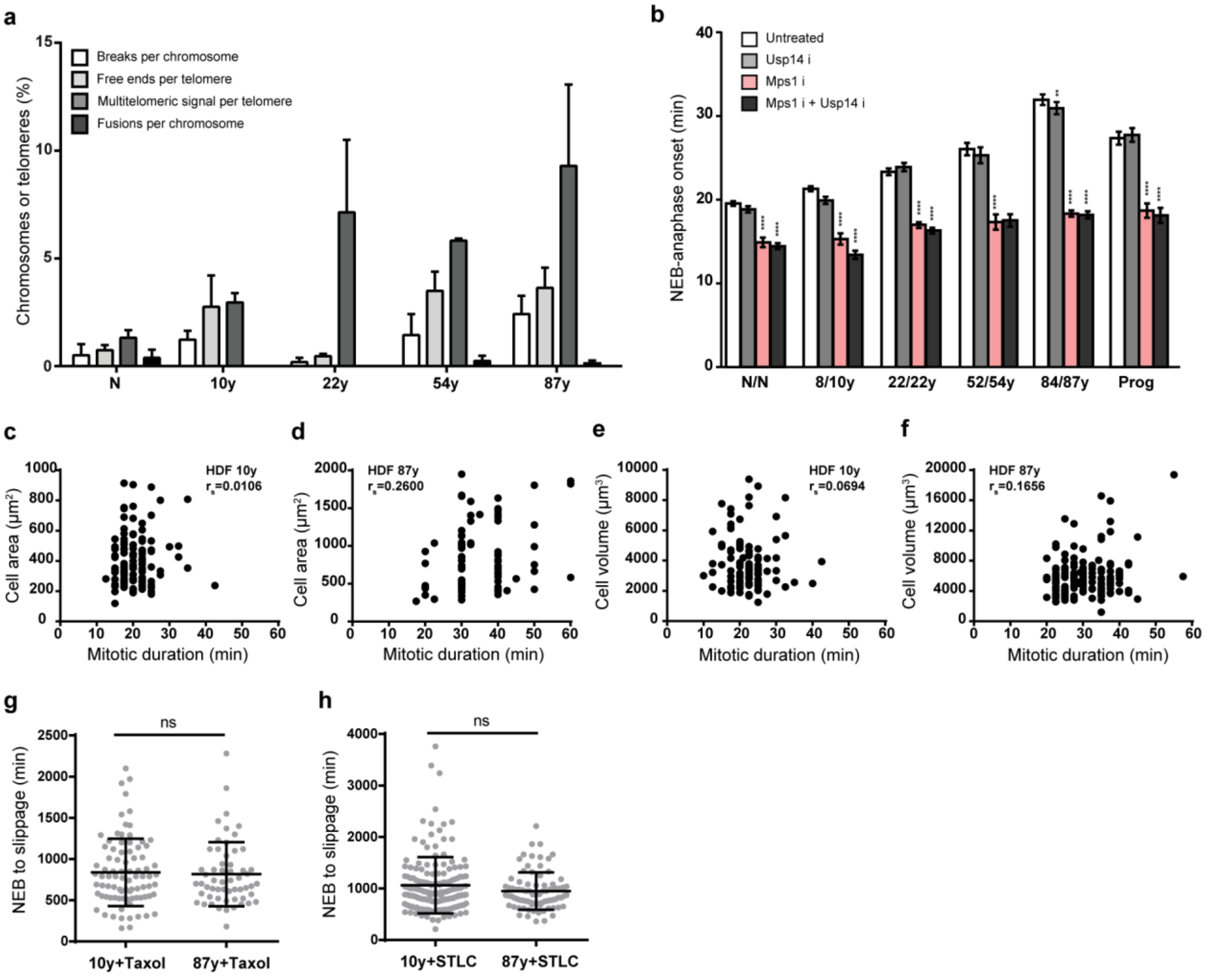

Supplementary Figure 3. Effects of telomere dysfunction, SAC activity, proteotoxic stress and cell size in mitotic duration. **a** Metaphase spreads of colcemid-treated cells of different age donors were quantified for chromosome fusions, chromosome breaks, telomere free ends (markers of telomere crisis) and multitelomeric signals (MTS; marker of telomere replication stress). At least 900 chromosomes or 3.500 telomeres were quantified in two independent experiments. Values are shown as mean ± s.e.m. Age-dependent significant differences in chromosome aberrations were tested by one-way ANOVA for linear trend and multiple comparisons. Chromosome fusions, chromosome breaks and telomere free ends, not significant; MTS, linear trend with p-value≤0.05. **b** Mitotic duration of human fibroblasts from different age donors cultured under standard conditions (white bars) and following treatment with Usp14 inhibitor (light gray bars), Mps1 inhibitor (colored bars) and

Mps1+Usp14 inhibitors (dark gray bars). **c-f** Spearman's correlation coefficients (r_s) between mitotic duration and **c-d** cell area or **e-f** cell volume. **g-h** Elapsed time from nuclear envelope breakdown (NEB) to cell's adherence into growth surface (slippage) in fibroblasts treated with the spindle poisons **g** taxol and **h** kinesin-5 inhibitor (STLC). **b-h** Values are mean ± s.d. of n>50 scored cells from three independent experiments. ns: $p>0.05$, **$p\leq0.01$, ***$p\leq0.001$ by Mann-Whitney statistical test.

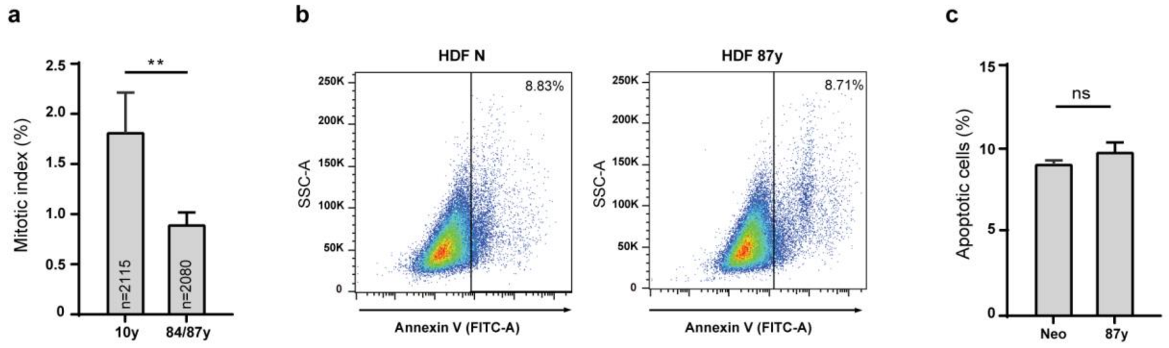

Supplementary Figure 4. Proliferation and apoptosis rate in aged HDFs. **a** Mitotic indices in young (HDF 10y) and elderly (HDF 87y) cell populations (n, sample size). **b** Flow cytometry analysis of Annexin V-FITC apoptosis marker in young (HDF N) and elderly (HDF 87y) cell populations. The gates were defined accordingly to the respective auto-fluorescent control. **c** Percentage of apoptotic cells (Annexin V-positive). Values in graphs are mean ± s.d. from two independent experiments. ns: $p>0.05$ and $**p\leq0.01$ by two-tailed χ^2 statistical test.

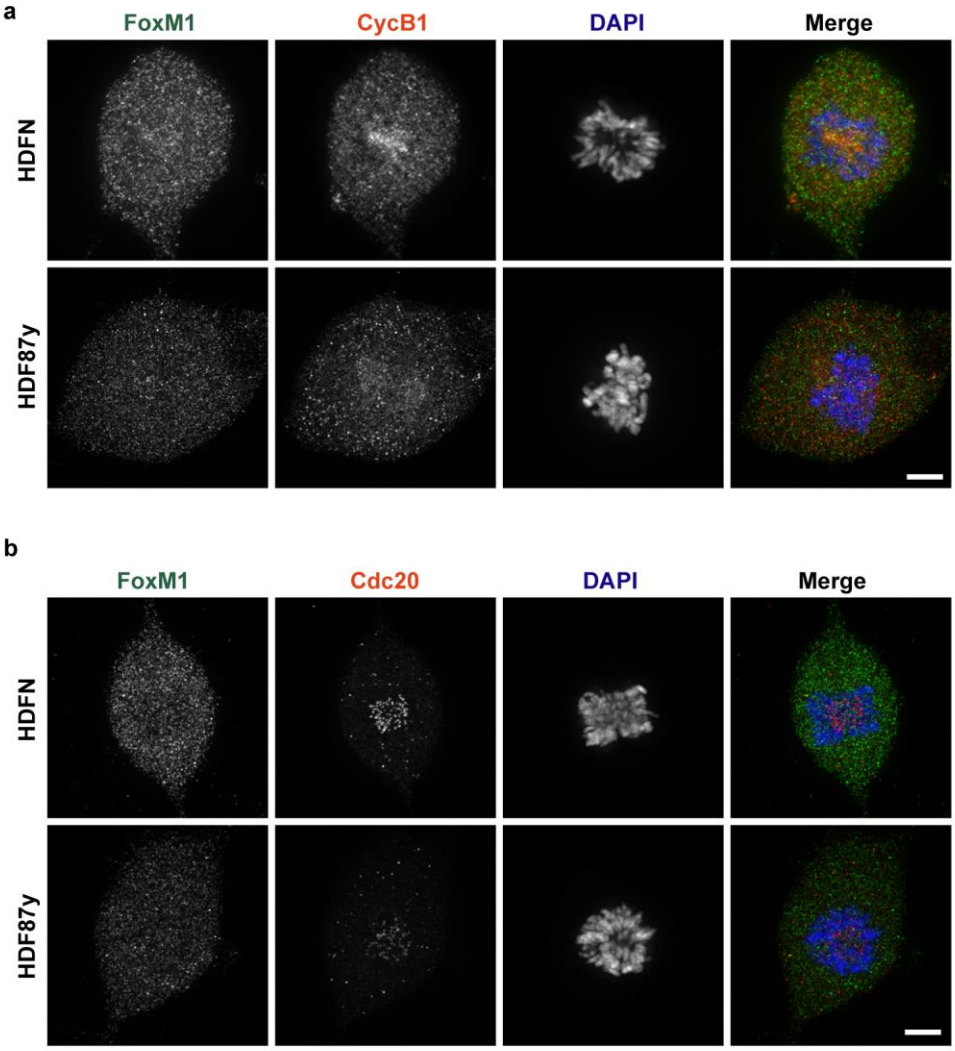

Supplementary Figure 5. Low levels of FoxM1 and mitotic gene targets in aging cells during mitosis. **a** FoxM1 and Cyclin B1 immunofluorescence levels in neonatal (HDFN) and octogenarian (HDF87y) mitotic fibroblasts. **b** FoxM1 and Cdc20 immunofluorescence levels in neonatal (HDFN) and octogenarian (HDF87y) mitotic fibroblasts. Scale bar, 5 µm. Supplementary figure to Fig. 4h-j.

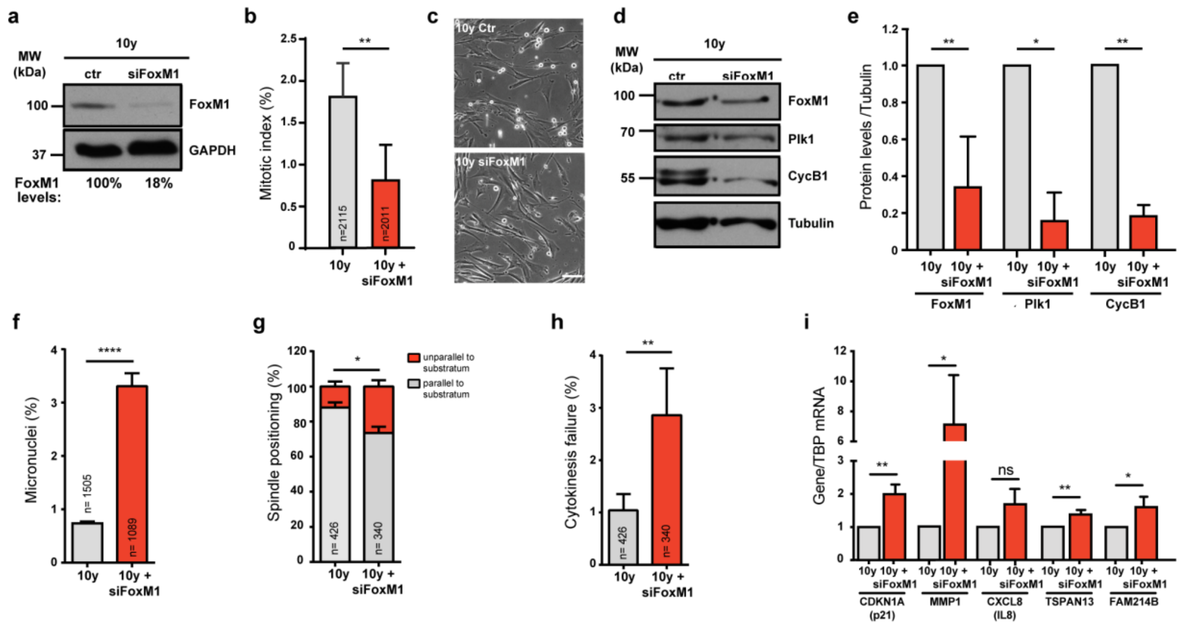

Supplementary Figure 6. FoxM1 repression in 10 year-old fibroblasts. **a** Western blot analysis of FoxM1 protein levels following RNAi depletion. GAPDH was used as loading control. **b** FoxM1 RNAi depletion (siFoxM1) decreases mitotic index. **c** Representative phase-contrast images of control- and siFoxM1-depleted 10y fibroblasts treated with STLC for mitotic enrichment and experimental layout shown in Fig. 3a. **d-e** Western blot analysis of FoxM1, Plk1 and Cyclin B1 protein levels in mitotic cell extracts of 10y fibroblasts upon FoxM1 RNAi depletion. Tubulin was used as loading control. **f-h** Mitotic phenotypes in siFoxM1-depleted 10y fibroblasts. **f** Percentage of cells with micronuclei. **g** Percentage of mitotic cells exhibiting unparallel spindle to the substratum. **h** Percentage of mitotic cells failing cytokinesis. **i** Transcript levels of senescence-associated genes in FoxM1-depleted young cells normalized to *TBP* transcript levels and compared to control cells. Scale bar, 100 µm. Values are mean ± s.d. from two (e, i) or three (b, f-h) independent experiments. Sample size (n) is indicated in each graph. *$p \leq 0.05$, **$p \leq 0.01$, ***$p \leq 0.001$ and ****$p \leq 0.0001$ by Mann-Whitney (b, e, i) and two-tailed χ^2 (f-h) statistical tests. Supplementary figure to Fig. 5.

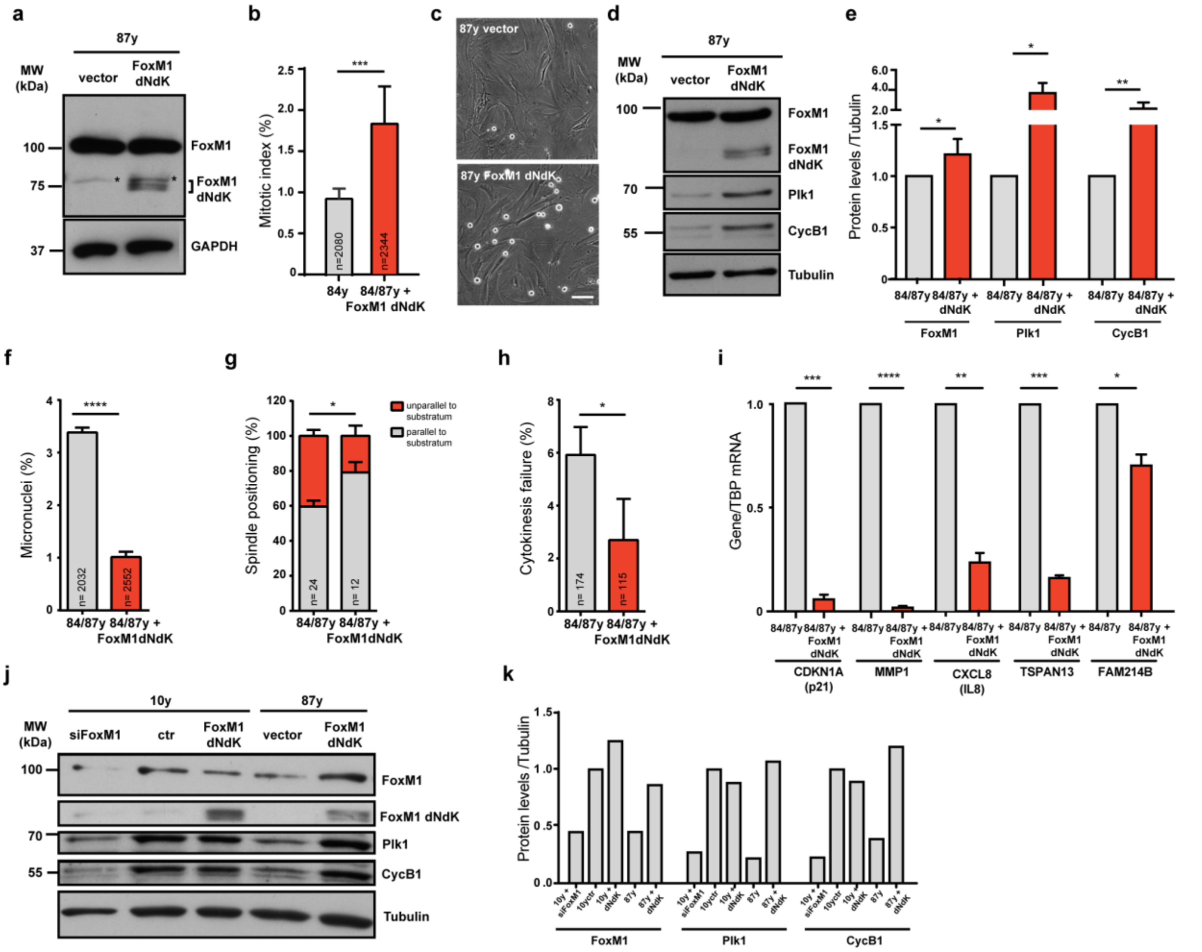

Supplementary Figure 7. Expression of constitutively active FoxM1 (FoxM1dNdK) in elderly cells. **a** Western blot analysis of FoxM1dNdK expression. Asterisk indicates an unspecific band. GAPDH was used as loading control. **b** FoxM1dNdK expression increases mitotic index. **c** Representative phase-contrast images of 87y fibroblasts transduced with empty vector or FoxM1dNdK lentiviruses and treated with STLC for mitotic enrichment and experimental layout shown in Fig. 3a. **d-e** Western blot analysis of FoxM1, Plk1 and Cyclin B1 protein levels in mitotic cell extracts of 87y fibroblasts expressing FoxM1dNdK. Tubulin was used as loading control. **f-h** Mitotic phenotypes in 84/87y fibroblasts expressing FoxM1dNdK. **f** Percentage of cells with micronuclei. **g** Percentage of mitotic cells exhibiting unparallel spindle to the substratum (in red). **h** Percentage of mitotic cells failing cytokinesis. **i** Transcript levels of senescence-associated genes in FoxM1dNdK elderly cells normalized to

TBP transcript levels and compared to control elderly cells. **j** Western blot comparative analysis of FoxM1 and its mitotic targets, Plk1 and Cyclin B1, in 10y vs. 87y fibroblasts under different experimental conditions as indicated. **k** Quantification of FoxM1, Plk1 and Cyclin B1 protein levels in the western blot shown in j), normalized to tubulin levels and compared to control (ctr) 10y sample. Scale bar, 100 µm. Values are mean ± s.d. from two (b, i) or three (e-h) independent experiments. Sample size (n) is indicated in each graph. *$p \leq 0.05$, **$p \leq 0.01$, ***$p \leq 0.001$ and ****$p \leq 0.0001$ by Mann-Whitney (b, e, i) and two-tailed χ^2 (f-h) statistical tests. Supplementary figure to Fig.6.

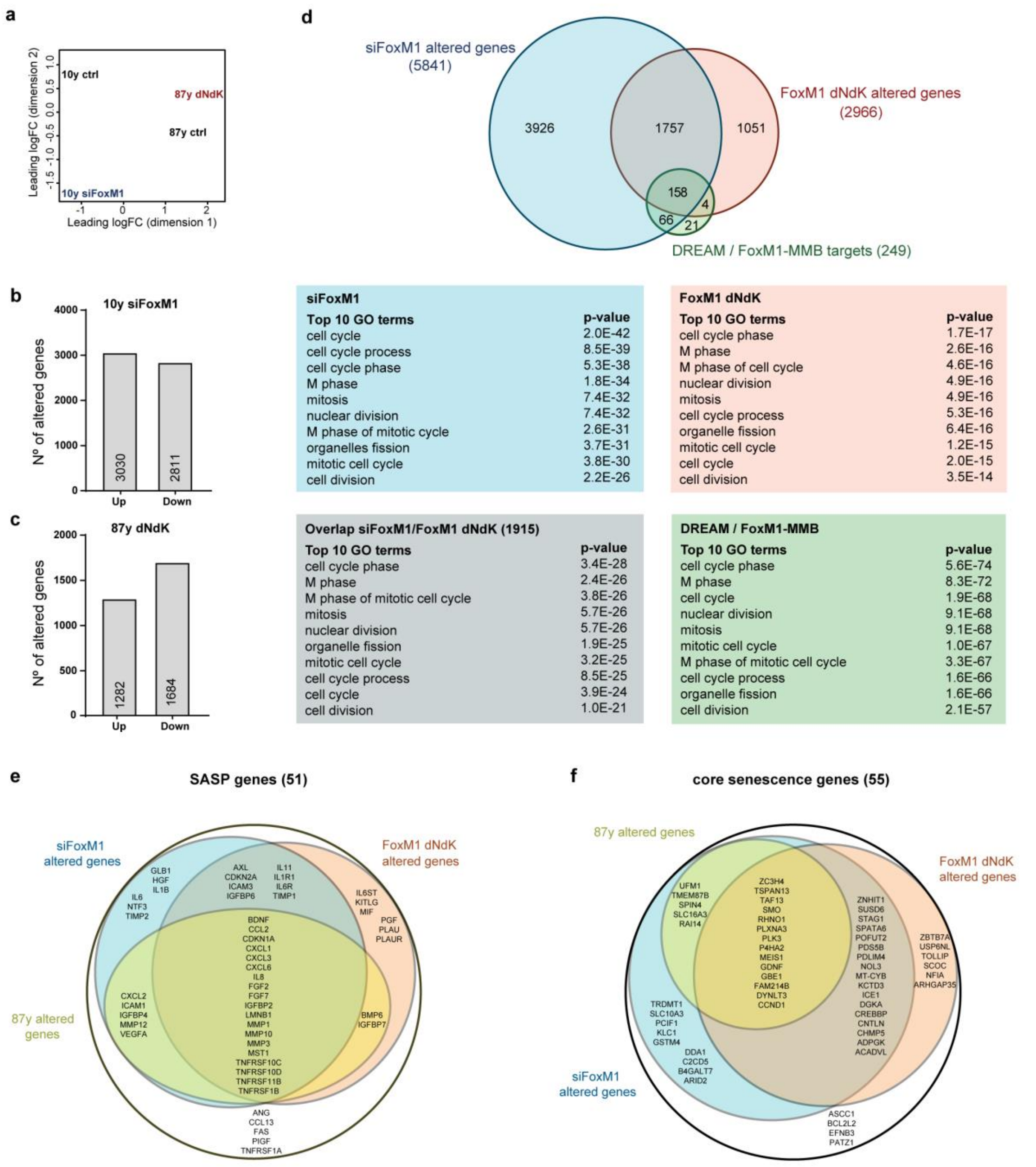

Supplementary Figure 8. Meta-analysis of differentially expressed genes upon modulation of FoxM1 levels. **a** Multidimensional scaling (MDS) plot of RNA-sequencing samples. Axes in the MDS plot represent leading log-fold changes (logFCs) calculated by the root-mean-square of the largest absolute logFCs between each pair of libraries for each sample. **b** Number of altered genes upon FoxM1 RNAi in 10y fibroblasts (Supplementary Data 7). **c** Number of altered genes upon lentiviral transduction of 87y cells with FoxM1dNdK

(Supplementary Data 12). **d** Venn diagram displaying the overlaps between siFoxM1 altered genes, FoxM1dNdK altered genes and targets of the DREAM/FoxM1-MMB complexes [41] (Supplementary Data 17). The top 10 GO terms for each overlap are organized by *p*-values, using the DAVID Functional Annotation tool. **e** Venn diagram displaying the overlaps between 51 SASP genes interrogated from the RNA-seq datasets of HDF 87y, 10y siFoxM1 and 87y FoxM1dNdK (Supplementary Data 4, 10 and 15). **f** Venn diagram displaying the overlaps between 55 genes of the "senescence core signature" [37] interrogated from the RNA-seq datasets of HDF 87y, 10y siFoxM1 and 87y FoxM1dNdK (Supplementary Data 5, 11 and 16).

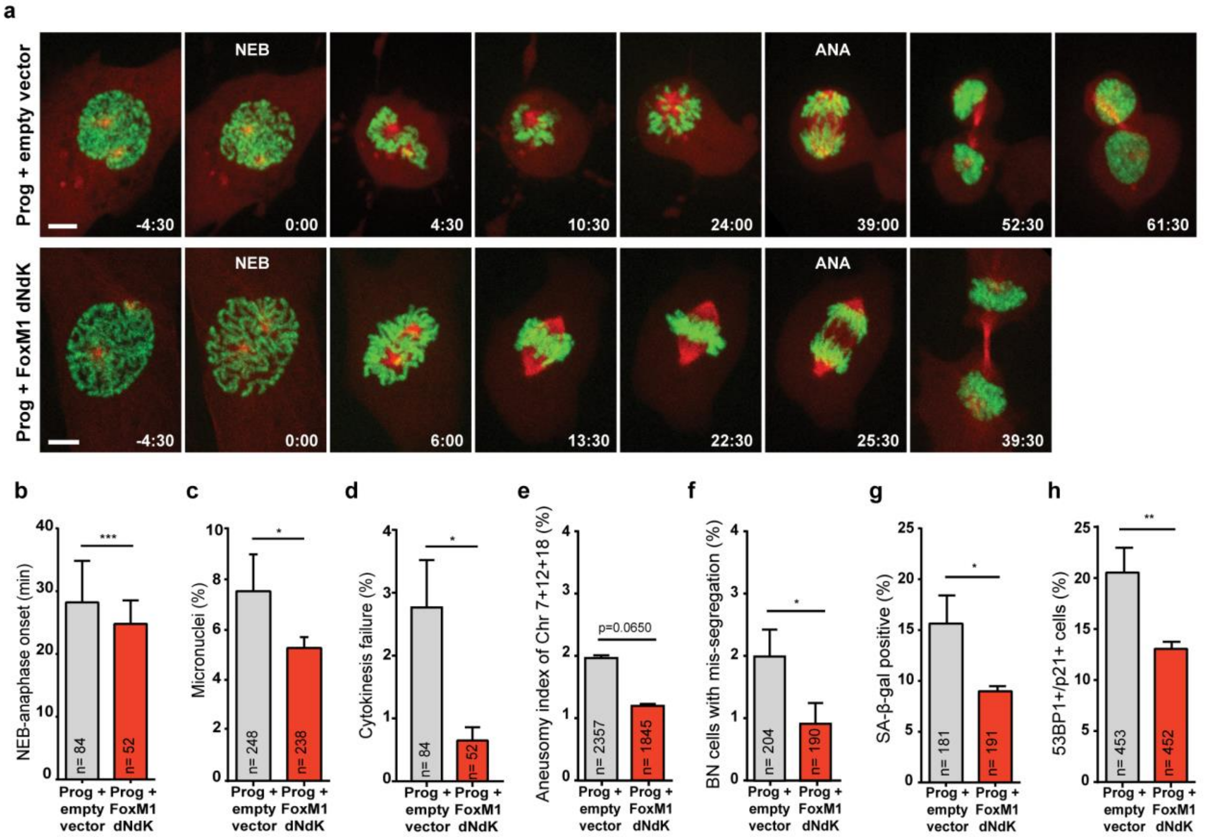

Supplementary Figure 9. Constitutively active FoxM1 ameliorates mitotic fitness and senescence markers in HGPS cells. **a** Movie frames of mitotic HGPS fibroblasts expressing H2B-GFP/α-Tubulin-mCherry (upper panel) and H2B-GFP/α-Tubulin-mCherry+FoxM1dNdK (lower panel) (Supplementary Movies 7, 8). NEB, nuclear envelope breakdown. ANA, anaphase onset. Time min:sec. Scale bar, 5 µm. **b** Mitotic duration of progeroid fibroblasts transduced with empty and FoxM1dNdK lentiviral vectors. **c** Percentage of cells with micronucleus. **d** Percentage of cells failing cytokinesis. **e** Aneusomy index in interphase FISH and **f** chromosome mis-segregation rate in Cyto D-FISH using centromeric probes against 3 chromosome pairs (7, 12 and 18). **g-h** Percentage of cells staining positive for the senescence markers **g** β-galactosidase and **h** 53BP1/p2l. Values are mean ± s.d. from at least two independent experiments. Sample size (n) is indicated in each

graph. *$p \leq 0.05$, **$p \leq 0.01$, ***$p \leq 0.001$ and ****$p \leq 0.0001$ by Mann-Whitney (b) and two-tailed $\chi 2$ (c-h) statistical tests.

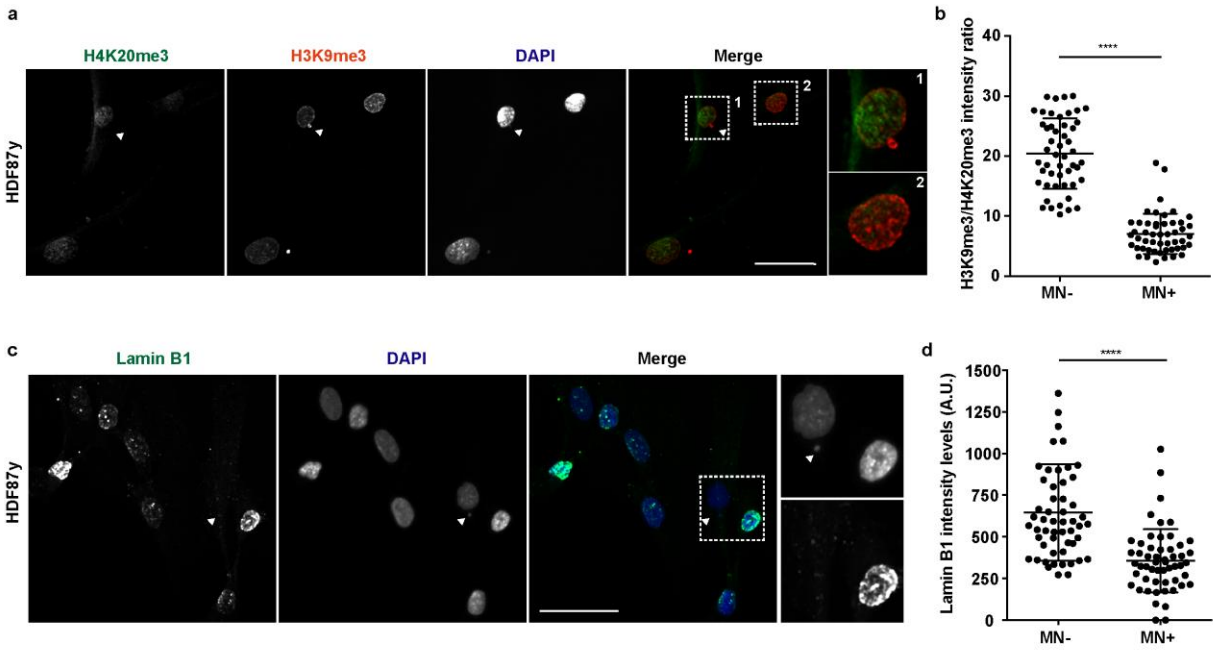

Supplementary Figure 10. Senescence markers in elderly fibroblasts with segregation defects. a Representative images of elderly cells (HDF87y) with segregation defects (presence of micronucleus, MN+) (arrowhead) and without segregation defects (MN-) stained for H4K20me3 and H3K9me3 epigenetic markers. Insets are 2x magnifications. **b** Quantification of H3K9me3/H4K20me3 intensity levels ratio. **c** Representative images of elderly cells (HDF87y) with (arrowhead) and without segregation defects stained for Lamin B1. Insets are 2x magnifications. **d** Quantification of Lamin B1 intensity levels. Scale bars, 50 µm. Scatter plots show n=50 cells for each condition. In all graphs, bars represent mean ± s.d. values from two independent experiments. ****$p \leq 0.0001$ by Mann-Whitney statistical test.

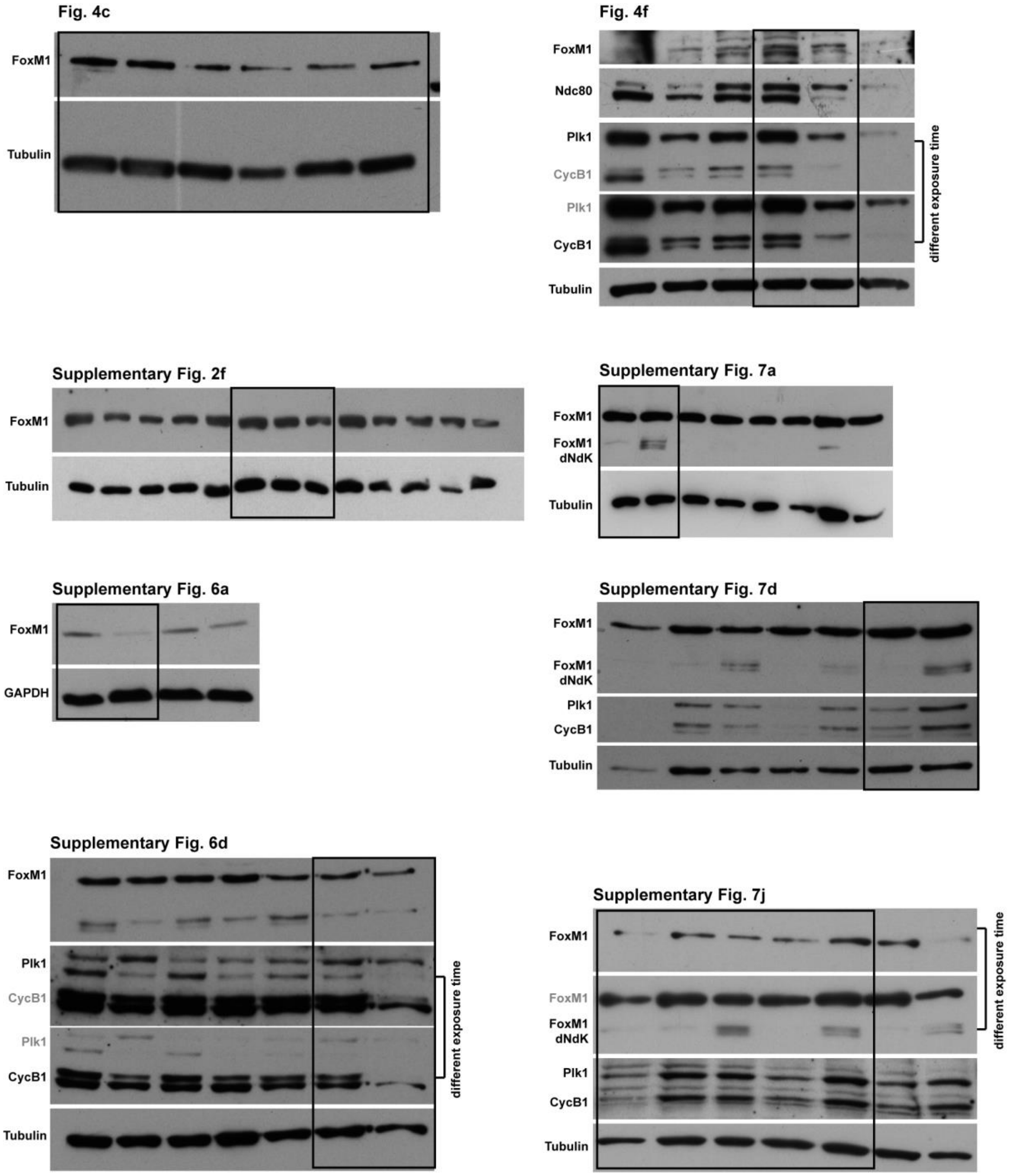

Supplementary Figure 11. Uncropped and unprocessed images of the Western blot results shown in Fig. 4c, Fig. 4f, Supplementary Fig. 2f, Supplementary Fig. 6a, Supplementary Fig. 6d, Supplementary Fig. 7a, Supplementary Fig. 7d and Supplementary Fig. 7j. Different exposure times of the same blot are indicated, as in some cases different proteins detected on the same blot were quantified under distinct exposure times.
