# Supplementary material for: FoxM1 repression during human aging leads to mitotic decline and aneuploidy-driven full senescence
Source: Nat Commun. 2018 Jul 19;9:2834. doi: 10.1038/s41467-018-05258-6 (PMC6053425; doi:10.1038/s41467-018-05258-6)
Supplement: Supplementary file 3 — Description of Additional Supplementary Files [file 41467_2018_5258_MOESM3_ESM.pdf]

## Description of Additional Supplementary Files

**File Name: Supplementary Movie 1**
**Description:** Mitotic progression of a neonatal fibroblast (HDF N) co-expressing H2B-GFP (green) and α-tubulin-mCherry (red). Time-lapse images were acquired on a spinning-disk confocal microscope every 90 sec. Time min:sec. Frame series from this movie were used in Fig. 2a.

**File Name: Supplementary Movie 2**
**Description:** Chromosome congression delay in a dividing elderly fibroblast (HDF 87y) co-expressing H2B-GFP (green) and α-tubulin-mCherry (red). Time-lapse images were acquired on a spinning-disk confocal microscope every 90 sec. Time min:sec. Frame series from this movie were used in Fig. 2b.

**File Name: Supplementary Movie 3**
**Description:** Chromosome anaphase lagging and micronucleus formation in a dividing elderly fibroblast (HDF 87y) co-expressing H2B-GFP (green) and α-tubulin-mCherry (red). Time-lapse images were acquired on a spinning-disk confocal microscope every 90 sec. Time min:sec. Frame series from this movie were used in Fig. 2c.

**File Name: Supplementary Movie 4**
**Description:** Unparallel mitotic spindle positioning in relation to the growth surface and asynchronous adherence of the daughter cells in a dividing elderly fibroblast (HDF 87y) co-expressing H2B-GFP (green) and α-tubulin-mCherry (red). Time-lapse images were acquired on a spinning-disk confocal microscope every 90 sec. Time min:sec. Frame series from this movie were used in Fig. 2d.

**File Name: Supplementary Movie 5**
**Description:** Mitotic delay from nuclear envelope breakdown (NEB) to anaphase onset (ANA) in an elderly fibroblast (HDF 87y) co-expressing H2B-GFP (green) and α-tubulin-mCherry (red). Time-lapse images were acquired on a spinning-disk confocal microscope every 90 sec. Time min:sec. Frame series from this movie were used in Fig. 6a.

**File Name: Supplementary Movie 6**
**Description:** Mitotic efficiency rescue following expression of FoxM1dNdK in an elderly fibroblast (HDF 87y) co-expressing H2B-GFP (green), α-tubulin-mCherry (red). Time-lapse images were acquired on a spinning-disk confocal microscope every 90 sec. Time min:sec. Frame series from this movie were used in Fig. 6a.

**File Name: Supplementary Movie 7**
**Description:** Chromosome alignment delay and mitotic spindle mispositioning in a HGPS (Progeria) fibroblast co-expressing H2B-GFP (green) and α-tubulin-mCherry (red). Time-lapse images were acquired on a spinning-disk confocal microscope every 90 sec. Time min:sec. Frame series from this movie were used in Supplementary Fig. 9a.

**File Name: Supplementary Movie 8**
**Description:** Mitotic efficiency rescue following FoxM1dNdK expression in a HGPS (Progeria) fibroblast co-expressing H2B-GFP (green) and α-tubulin-mCherry (red). Time-lapse images were acquired on a spinning-disk confocal microscope every 90 sec. Time min:sec. Frame series from this movie were used in Supplementary Fig. 9a.

**File Name: Supplementary Movie 9**
**Description:** Daughter cell fate tracking following cell division of an 87y fibroblast expressing H2B-GFP. Time-lapse images were acquired on a widefield inverted microscope with a 20xLD/NA0.4 dry objective every 5 min for 72h. In this movie, the mother cell divided normally generating daughter cells that kept on cycling (only one daughter cell is shown (*)). Frame series from this movie were used in Fig. 7h (upper panel). White lines represent the stitching of adjacent imaging fields.

**File Name: Supplementary Movie 10**
**Description:** Daughter cell fate tracking following cell division of an 87y fibroblast expressing H2B-GFP. Time-lapse images were acquired on a widefield inverted microscope with a 20xLD/NA0.4 dry objective every 5 min for 72h. In this movie, the mother cell divided without apparent chromosome mis-segregation generating daughter cells that stopped cycling (only one daughter cell is shown (*)). Frame series from this movie were used in Fig. 7h (middle panel). The last frame of the movie shows the correlative fixed-cell analysis of senescence biomarkers SA-β-gal (green), p21 (blue) and 53BP1 (red), with the tracked daughter cell staining negative for SA-β-gal activity and double 53BP1/p21. Neighboring cells are enumerated.

**File Name: Supplementary Movie 11**
**Description:** Daughter cell fate tracking following cell division of an 87y fibroblast expressing H2B-GFP. Time-lapse images were acquired on a widefield inverted microscope with a 20xLD/NA0.4 dry objective every 5 min for 72h. In this movie, chromosome mis-segregation occurred in the mother cell mitosis generating daughter cells with micronuclei that stopped cycling and stained positive for senescence biomarkers (only one daughter cell is shown (*)). Frame series from this movie were used in Fig. 7h (lower panel). White line represents the stitching of adjacent imaging fields. The last frame of the movie shows the correlative fixed-cell analysis of SA-β-gal (green), p21 (blue) and 53BP1 (red), with the tracked daughter cell staining positive for all senescence biomarkers. Neighboring cells are enumerated.

**File Name: Supplementary Data 1**
**Description:** RNA-sequencing dataset of differentially expressed genes in neonatal vs. 87y mitotic fibroblasts. Two technical replicates were analysed for each biological sample (Materials and Methods). Genes with 2logFC>0.5 or <-0.5, and *p*-value<0.05, were considered significantly altered. The dataset supports Fig. 3d-f.

**File Name: Supplementary Data 2**
**Description**: RNA-sequencing dataset of differentially expressed 'Mitosis' GO term genes in neonatal vs. 87y mitotic fibroblasts. Two technical replicates were analysed for each biological sample (Materials and Methods). Genes with 2logFC>0.5 or <-0.5, and *p*-value<0.05, were considered significantly altered. The dataset supports Fig. 3g.

**File Name: Supplementary Data 3**
**Description:** Rationale behind the comprehensive list of 51 SASP (chemokines, cytokines, metalloproteinases and others) and senescence-associated genes (*CDKN1A, CDKN2A, GLB1,*

*LMNB1)* interrogated from the RNA-sequencing datasets. The list includes gene products known as oversecreted in conditioned medium of senescent fibroblasts [36], merged with genes known as altered in senescent vs. proliferating cells [37].

**File Name: Supplementary Data 4**
**Description:** SASP and senescence-associated genes differentially expressed in neonatal vs. 87y mitotic fibroblasts. In the RNA-sequencing dataset, genes with *p*-value<0.05 were considered significantly altered. 2logFCs in red indicate genes behaving differently from expected [36,37]. The dataset supports Fig. 3h.

**File Name: Supplementary Data 5**
**Description:** 'Senescence core signature' genes differentially expressed in neonatal vs. 87y mitotic fibroblasts. In the RNA-sequencing dataset, genes with *p*-value<0.05 were considered significantly altered. 2logFCs in red indicate genes behaving differently from expected [37]. The dataset supports Fig. 3i.

**File Name: Supplementary Data 6**
**Description:** Mitotic genes differentially expressed in neonatal vs. 87y mitotic fibroblasts reported as targets of the Myb-MuvB(MMB)-FoxM1 transcription complex. The dataset supports the venn diagram in Fig. 4b.

**File Name: Supplementary Data 7**
**Description:** RNA-sequencing datasets of differentially expressed genes in 10y vs. 10y siFoxM1-depleted mitotic fibroblasts. Two technical replicates were analysed for each experimental condition (Materials and Methods). Genes with 2logFC>0.5 or <-0.5, *p*-value<0.05 and FDR<0.05, were considered significantly altered. The dataset supports Supplementary Fig. 8a,b,d.

**File Name: Supplementary Data 8**
**Description:** RNA-sequencing datasets of differentially expressed 'Mitosis' GO term genes in 10y vs. 10y siFoxM1-depleted mitotic fibroblasts. Two technical replicates were analysed for each experimental condition (Materials and Methods). Genes with 2logFC>0.5 or <-0.5, *p*-value<0.05 and FDR<0.05, were considered significantly altered. The dataset supports Fig. 5a.

**File Name: Supplementary Data 9**
**Description:** List of genes differentially expressed in both 87y and 10y siFoxM1-depleted mitotic fibroblasts. The dataset supports the venn diagram in Fig. 5b.

**File Name: Supplementary Data 10**
**Description:** SASP and senescence-associated genes differentially expressed in 10y vs. 10y siFoxM1-depleted mitotic fibroblasts. In the RNA-sequencing dataset, genes with *p*-value<0.05 were considered significantly altered. The dataset supports Fig. 5h.

**File Name: Supplementary Data 11**
**Description:** 'Senescence core signature' genes differentially expressed in 10y vs. 10y siFoxM1-depleted mitotic fibroblasts. In the RNA-sequencing dataset, genes with *p*-value<0.05 were considered significantly altered. 2logFCs in red indicate genes behaving differently from expected [37]. The dataset supports Fig. 5i.

**File Name: Supplementary Data 12**
**Description:** RNA-sequencing dataset of diferentially expressed genes in 87y vs. 87y FoxM1dNdK-expressing mitotic fibroblasts. Two technical replicates were analysed for each experimental condition (Materials and Methods). Genes with 2logFC>0.5 or <-0.5, *p*-value<0.05 and FDR<0.05, were considered significantly altered. The dataset supports Supplementary Fig. 8a,c,d.

**File Name: Supplementary Data 13**
**Description**: RNA-sequencing dataset of differentially expressed 'Mitotic' GO term genes in 87y vs. 87y FoxM1dNdK-expressing mitotic fibroblasts. Two technical replicates were analysed for each experimental condition (Materials and Methods). Genes with 2logFC>0.5 or <-0.5, *p*-value<0.05, and FDR<0.05, were considered significantly altered. The dataset supports Fig.6b.

**File Name: Supplementary Data 14**
**Description:** Overlap between the 'Mitosis' GO term genes differentially expressed in 87y, 10y siFoxM1-depleted, and 87y FoxM1dNdK-expressing mitotic fibroblasts. The dataset supports the venn diagram in Fig. 6c.

**File Name: Supplementary Data 15**
**Description:** SASP and senescence-associated genes differentially expressed in 87y vs. 87y FoxM1dNdK-expressing mitotic fibroblasts. In the RNA-sequencing dataset, genes with *p*-value<0.05 were considered significantly altered. 2logFCs in red indicate genes behaving differently from expected [36,37]. The dataset supports Fig. 6i.

**File Name: Supplementary Data 16**
**Description:** 'Senescence core signature' genes differentially expressed in 87y vs. 87y FoxM1dNdK-expressing mitotic fibroblasts. In the RNA-sequencing dataset, genes with *p*-value<0.05 were considered significantly altered. 2logFCs in red indicate genes behaving differently from expected [37]. The dataset supports Fig. 6j.

**File Name: Supplementary Data 17**
**Description:** Overlap between differentially expressed genes in FoxM1 RNAi and FoxM1dNdK overexpression, and overlap with known DREAM/MMB-FoxM1 transcriptional targets. The dataset supports the venn diagram in Supplementary Fig. 8d.
